# Fatal pancreatic fistula after laparoscopic distal pancreatectomy for intraductal papillary mucinous carcinoma with pancreaticobiliary maljunction and sphincterotomized papilla: a case report

**DOI:** 10.1186/s40792-021-01324-2

**Published:** 2021-11-10

**Authors:** Yoshifumi Morita, Tomohiro Akutsu, Mitsumasa Makino, Miku Obayashi, Shinya Ida, Ryuta Muraki, Ryo Kitajima, Amane Hirotsu, Makoto Takeda, Hirotoshi Kikuchi, Yoshihiro Hirmatsu, Yasushi Hamaya, Ken Sugimoto, Hiromi Kato, Matsuyuki Doi, Yukichi Tanahashi, Satoshi Goshima, Takanori Sakaguchi, Hiroya Takeuchi

**Affiliations:** 1 Second Department of Surgery, Hamamatsu University School of Medicine, 1-20-1 Handayama, Higashi-ku, Hamamatsu, 431-3192 Japan; 2 Department of Perioperative Functioning Care and Support, Hamamatsu University School of Medicine, Hamamatsu, Japan; 3 Department of Gastroenterological Medicine, Hamamatsu University School of Medicine, Hamamatsu, Japan; 4 Department of Anesthesiology and Intensive Care Medicine, Hamamatsu University School of Medicine, Hamamatsu, Japan; 5 Department of Radiology, Hamamatsu University School of Medicine, Hamamatsu, Japan; 6 Department of Gastroenterological Surgery, Iwata City Hospital, Iwata, Japan

**Keywords:** Pancreaticobiliary maljunction, Distal pancreatectomy, Fatal pancreatic fistula, Intraductal papillary mucinous neoplasm, Endoscopic sphincterotomy

## Abstract

**Background:**

Pancreatic juice is constantly activated by contaminated bile in patients with pancreaticobiliary maljunction (PBM). Here, we report a case of laparoscopic distal pancreatectomy for a patient with PBM and sphincterotomized papilla, resulting in fatal pancreatic fistula.

**Case presentation:**

A 79-year-old man was diagnosed with pancreatic intraductal papillary mucinous neoplasm and common bile duct stones. Endoscopic sphincterotomy was performed prior to surgery. The pancreatic duct was simultaneously visualized when the contrast agent was injected into the common bile duct. Sudden bleeding was observed from the abdominal drain on postoperative day (POD) 6. Emergent stent graft placement and coil embolization were performed for bleeding from the splenic artery. On POD 9, the drainage fluid changed to yellowish in color with bile contamination. For internal drainage of the digestive fluid, endoscopic retrograde biliary tube and pancreatic drainage tube were placed. On POD 24, second emergent coil embolization was performed for bleeding from the left gastric artery. On POD 25, open abdominal drainage was performed. On POD 32, third emergent coil embolization was performed for bleeding from the gastroduodenal artery. Subsequently, remnant pancreatic resection was performed. On POD 39, massive bleeding was again observed from the abdominal drain. Emergency arterial portography revealed bleeding in the right wall of the superior mesenteric vein. The patient died of hemorrhagic shock on the same day.

**Conclusions:**

The extreme risk of severe pancreatic fistula after distal pancreatectomy should be considered in patients with PBM and sphincterotomized papilla. In this extraordinary situation, surgeons should promptly decide whether to resect the remnant pancreas to prevent losing the patient.

## Background

Pancreaticobiliary maljunction (PBM) is a rare congenital malformation in which the pancreatic and bile ducts join anatomically outside the duodenal wall [1]. PBM without

biliary dilatation often remains asymptomatic in adults. In patients with PBM, the pancreatic juice is constantly activated by contaminated bile. Here, we report a case of laparoscopic distal pancreatectomy for pancreatic intraductal papillary mucinous neoplasm (IPMN) with PBM and sphincterotomized papilla, resulting in repeated rupture of a pseudoaneurysm (PA) in the early postoperative period. Although we performed three times of endovascular interventions and two times of re-laparotomies, the patient eventually died of hemorrhagic shock.

## Case presentation

**Fig. 1:**
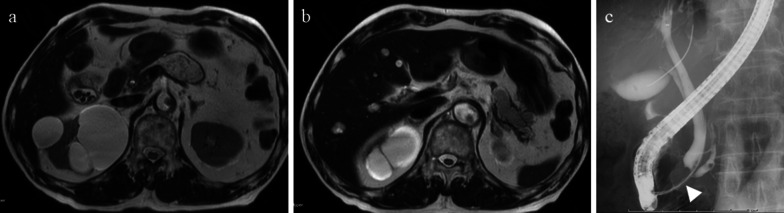
Preoperative imaging findings. **a** T2-weighted MRI showed a cystic tumor, 48 mm in diameter, in the pancreatic body. **b** Size of the main pancreatic duct was 18 mm in the pancreatic tail. **c** ERCP showed that the length of the common channel connecting the bile duct and pancreatic duct was 12 mm (arrowhead)

**Fig. 2:**
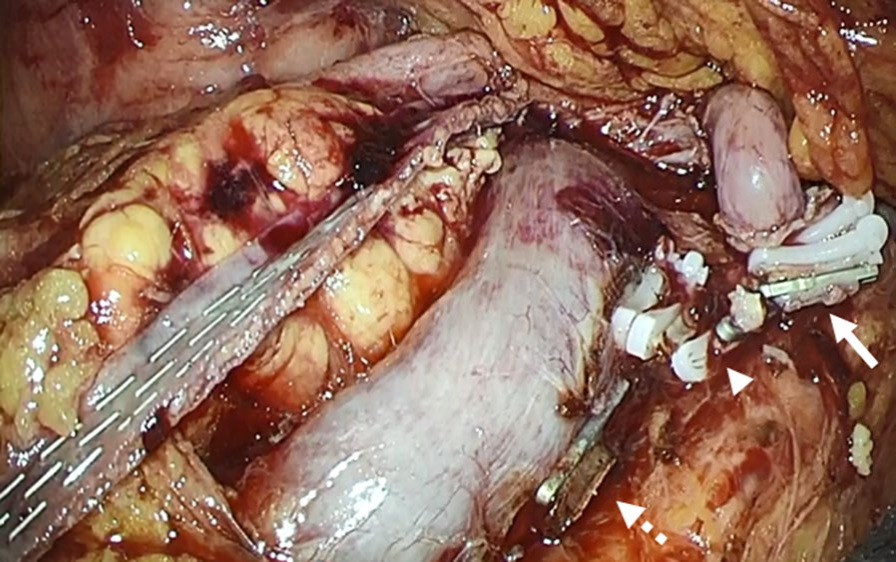
Intraoperative findings around the stump of the pancreas. The stump of the pancreas appeared to be well enclosed with a linear stapler. The stump of the splenic artery (arrow), splenic vein (arrowhead), and inferior mesenteric vein (dotted arrow)

**Fig. 3:**
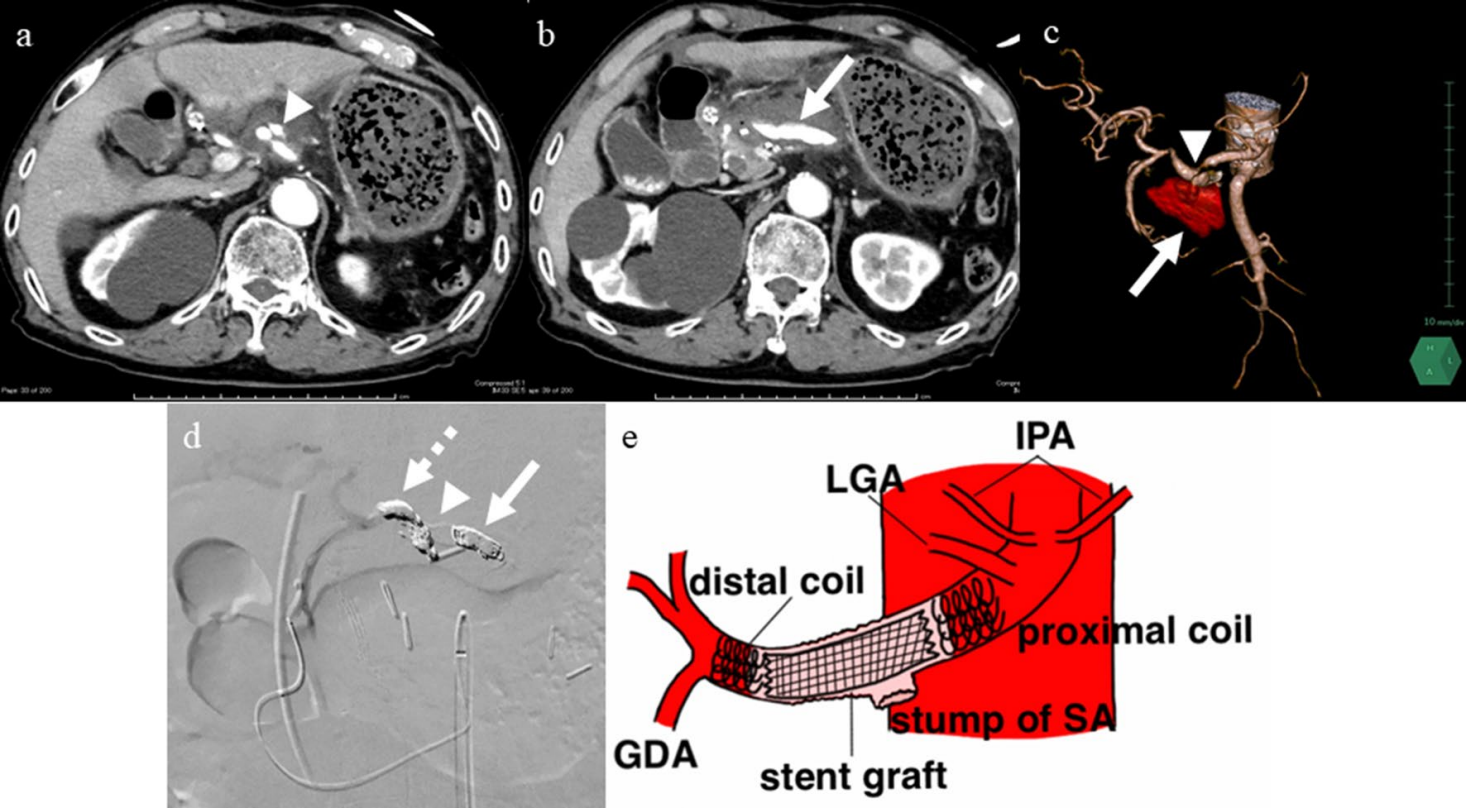
Imaging findings at postoperative day 6. **a** Enhanced CT showed a pseudoaneurysm at the stump of the splenic artery (arrowhead). **b** Enhanced CT showed extravasation of contrast medium (arrow). **c** Reconstruction image of the abdominal arteries and extravasation from the stump of the splenic artery (arrow). Stenosis and poststenotic dilatation of the common hepatic artery (arrowhead). **d** Superior mesenteric artery to the gastroduodenal artery angiography after placement of the stent graft (arrowhead), proximal coil (arrow), and distal coil (dotted arrow). **e** Schema after the placement of stent graft and coil embolization. *GDA* gastroduodenal artery, *LGA* left gastric artery, *IPA* infraphrenic artery, *SA* splenic artery

**Fig. 4:**
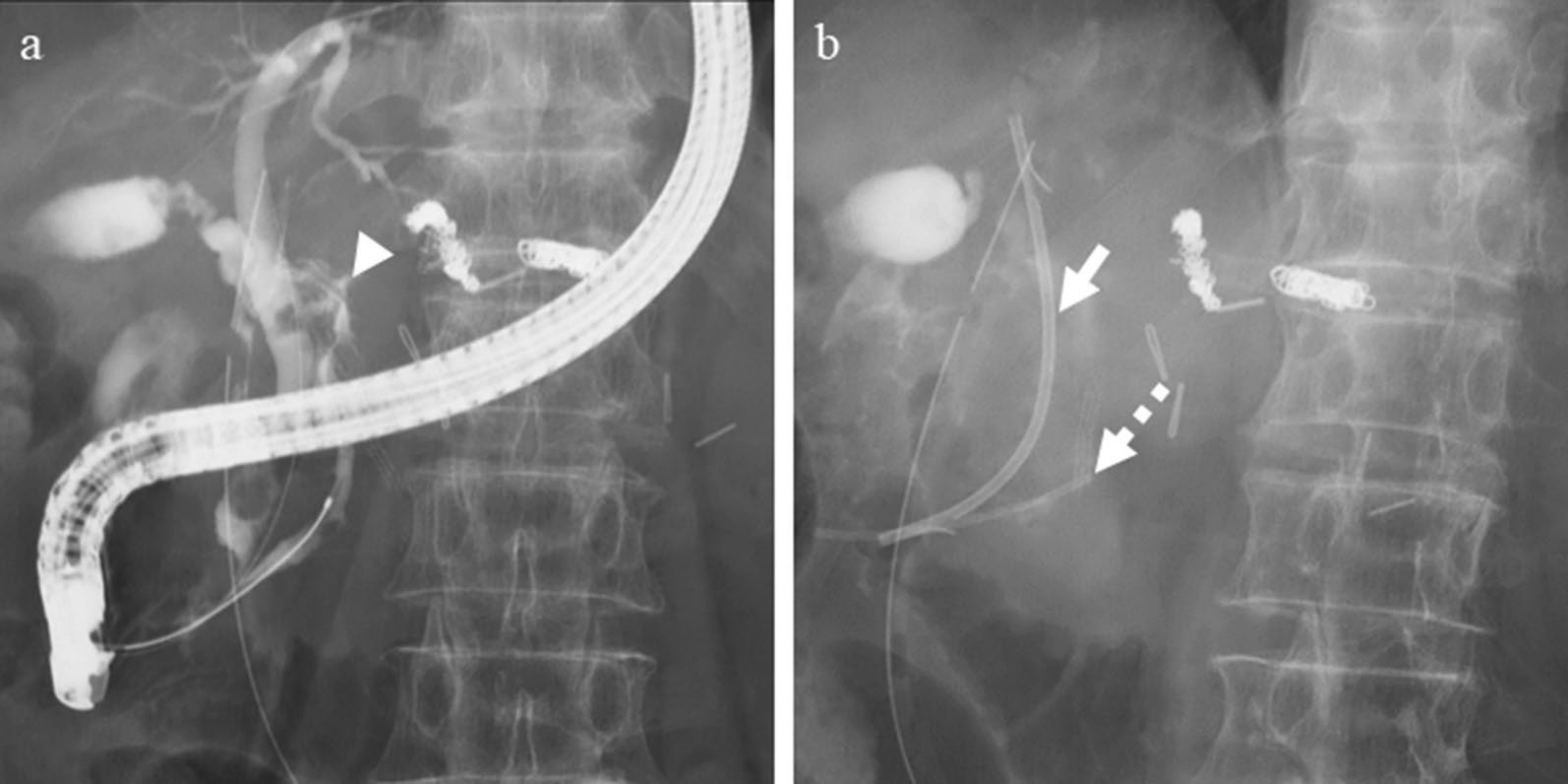
Endoscopic retrograde pancreatography at postoperative day 9. **a**, **b** Leakage from the main pancreatic duct (arrowhead), ERBD (arrow), and ERPD (dotted arrow)

**Fig. 5:**
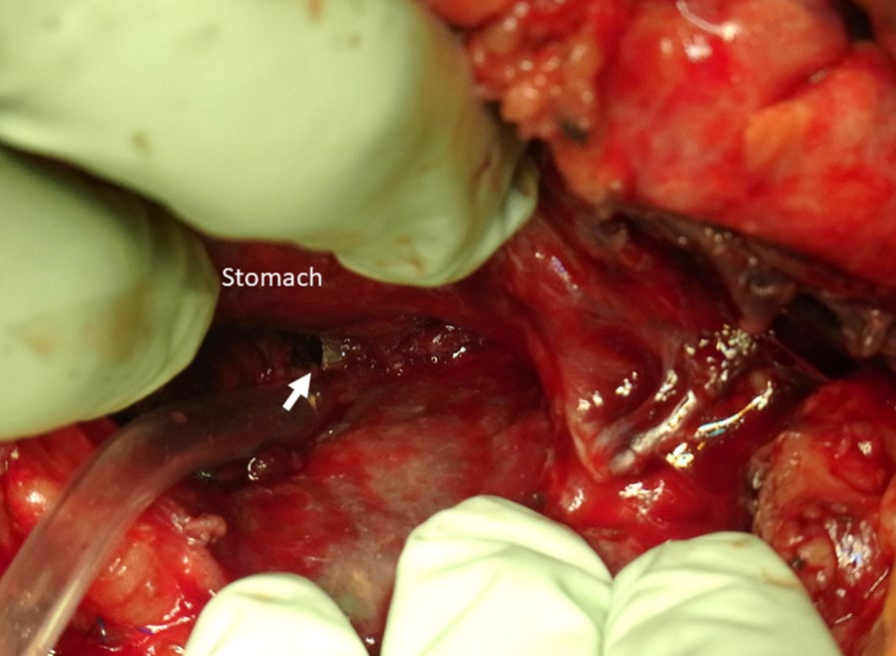
Intraoperative findings during the second operation. The Arterial wall of the common hepatic artery disappeared, and the stent graft (arrow) was exposed

**Fig. 6:**
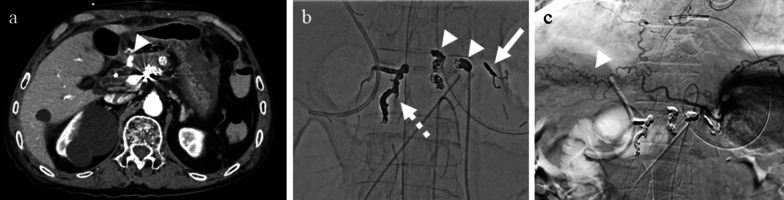
Imaging findings at postoperative day 32. **a** Enhanced CT showed pseudoaneurysm at the gastroduodenal artery (arrowhead). **b** Angiography showed the partially dislocated coil placed at the common hepatic artery (arrowhead), left gastric artery (arrow), and gastroduodenal artery (dotted arrow). **c** Angiography from the root of the celiac artery showed intrahepatic arterial flow (arrowhead) from the right inferior phrenic artery

**Fig. 7:**
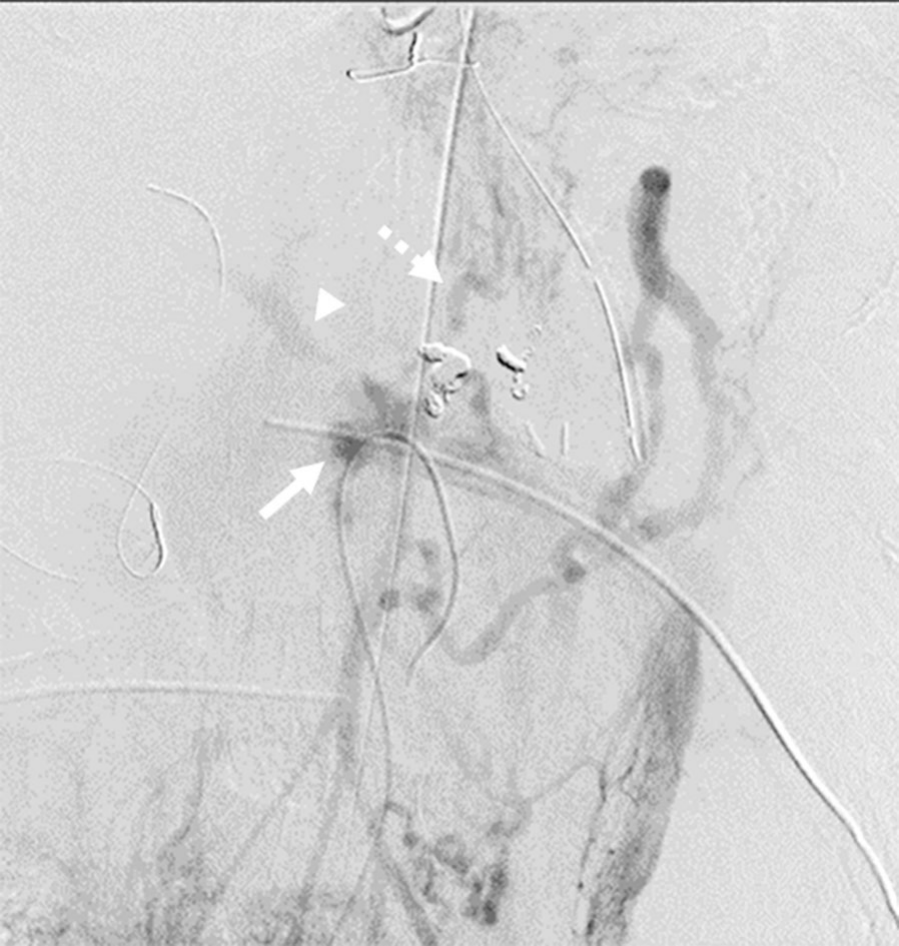
Arterial portography at postoperative day 39. Extravasation of contrast medium from the right side of the superior mesenteric vein (arrow), faint visualization of the portal vein (arrowhead), and left gastric vein (dotted arrow)

A 79-year-old man was diagnosed with pancreatic IPMN, gallstones, and common bile duct stones before surgery for aortic regurgitation, mitral regurgitation, and tricuspid regurgitation. Pancreatic IPMN worsened after cardiac surgery and the patient was referred to our department. Pancreatic IPMN showed high-risk stigmata of enlarged cyst and dilated main pancreatic duct of 48 mm and 18 mm in diameter, respectively (Fig. 1a, b). Prior to surgery, endoscopic sphincterotomy was performed for common bile duct stones. We found that the common channel connecting the bile duct and the pancreatic duct was relatively long at 12 mm in length. The pancreatic duct was simultaneously visualized when the contrast agent was injected into the common bile duct (Fig. 1c). Laparoscopic distal pancreatectomy and splenectomy were performed using a stapler with biological reinforcement. The intraoperative appearance of the stump of the pancreas appeared to be well enclosed (Fig. 2). The stump of the pancreas was covered with a polyglycolic acid mesh with fibrin glue. The operation time was 323 min, and the bleeding volume was 90 ml. Concomitant cholecystectomy was avoided to reduce operation time and physical burden. Although the drain amylase level at the stump of the pancreas on postoperative day (POD) 1 was elevated, it gradually decreased day by day after treatment with non-eating, antibiotic, and somatostatin analog (Table 1). Contrast-enhanced computed tomography (CT) on POD 4 showed no obvious PA formation (data not shown). Sudden bleeding was observed from the abdominal drain near the stump of the pancreas on POD 6 (Fig. 3a, b, c). Emergent stent graft placement. Emergent stent graft placement and coil embolization were performed for the ruptured PA from the stump of the splenic artery (SA) to the common hepatic artery (CHA). Initially, we placed a stent graft (φ 6 mm × 25 mm, GORE^®^ VIABAHN^®^) extending from the celiac artery (CA) to the CHA. However, extravasation from the SA persisted even after stent-graft placement, and we thought that this was because the stent graft was smaller than the CA. Next, we performed coil embolization at the proximal side of the stent graft. Then, we performed additional distal coil embolization from the superior mesenteric artery (SMA). The ruptured artery was completely isolated from the systemic circulation, and hepatic arterial flow was maintained from the SMA to the gastroduodenal artery (GDA) (Fig. 3d, e). The aspartate aminotransferase (AST) and alanine aminotransferase (ALT) levels were not elevated after the placement of stent graft and coil embolization. We then replaced the abdominal drain and initiated continuous irrigation and suction of saline. On POD 9, the drainage fluid changed to yellowish in color with bile contamination (Table 1). Endoscopic retrograde pancreatography confirmed leakage from the main pancreatic duct. Endoscopic retrograde biliary drainage (ERBD) tube and endoscopic retrograde pancreatic drainage (ERPD) tube were placed as internal drainage of the digestive fluid (Fig. 4). On POD 24, second emergent coil embolization was performed for bleeding from the periphery of the left gastric artery (data not shown). On POD 25, open abdominal drainage, omental filling around the pancreatic stump, cholecystectomy, and tube ileostomy were performed. The second operation time was 252 min, and the bleeding volume was 1090 ml. The second operation revealed that the arterial wall of the CHA had disappeared, and the stent graft was exposed (Fig. 5). On POD 32, third emergent coil embolization was performed for the ruptured PA at the gastroduodenal artery (Fig. 6a, b). Intrahepatic arterial flow was maintained from the right inferior phrenic artery (Fig. 6c). A balloon catheter was placed at the root of the celiac artery. Subsequently, remnant pancreatic resection was performed. The stent graft placed at the CHA was removed under balloon occlusion of the celiac artery. During the operation, massive bleeding occurred from the right side of the portal vein and was controlled by continuous sutures. The third operation time was 506 min, and the bleeding volume was 5372 ml. After total pancreatectomy, the patient developed renal failure, liver failure, and sepsis. The AST and ALT levels after the third operation were 1662 U/L and 867 U/L, respectively. On POD 39, massive bleeding was again observed from the abdominal drain. Emergent arterial portography under occlusion of the intra-aortic balloon revealed stenosis of the portal vein sutured during the third operation. The bleeding point was the right wall of the superior mesenteric vein, where the remnant pancreas was removed (Fig. 7). Although we attempted a percutaneous transhepatic approach, it was unable to treat due to poor blood flow in the intrahepatic portal vein. The patient died of hemorrhagic shock on the same day.

**Table 1:** Postoperative biological and culture data

	POD 1	POD 2	POD 3	POD 4	POD 7	POD 9	POD 14	POD 24	POD 26	POD 28	POD 30
B.T (℃)	37.0	37.8	37.5	37.0	37.3	37.2	37.6	37.8	38.4	37.2	37.1
CRP (mg/dl)	3.17	13.03	25.43	21.86	7.71	10.40	5.74	3.99	7.54	17.60	4.85
WBC (/μl)	11,980	17,530	15,380	9550	15,190	10,100	13,260	12,600	8470	9500	7710
Drain AMY (U/L)	17,623	10,447	4562	5243	115	124,999	168,010	378	116,760	191,584	248,090
Drain bil (mg/dl)	N.A	N.A	N.A	N.A	N.A	4.6	0.1	N.A	0.4	0.1	0.1
Drain culture	N.A	N.A	Negative	N.A	N.A	Enterococcus	Corynebacterium, Pseudomonas, Candida	N.A	N.A	MRSA, Candida	N.A

*N.A.* not assessed

## Discussion

Pancreaticobiliary maljunction (PBM) was first reported in the Japanese literature by Kozumi et al. in 1916 [2]. In the English literature, Babbitt first reported three patients with an anomalous arrangement of the pancreaticobiliary ductal system in 1969 [3]. PBM is a congenital anomaly in which the pancreatic and bile ducts join anatomically outside the duodenal wall. According to the Japanese clinical practice guidelines for pancreaticobiliary maljunction, PBM includes one type that is associated with bile duct dilatation (congenital biliary dilatation), and another without biliary dilatation [4]. When the sphincter of Oddi contracts, pancreatic juice flows back into the common bile duct, causing chronic inflammation of the biliary tract. Both types of PBM are associated with a high incidence of biliary tract cancer. PBM without biliary dilatation often remains asymptomatic, even in adults. However, the reciprocal reflux of pancreatic juice and bile are remain persistent. Pancreatic enzymes, particularly phospholipase A_2_, are occasionally activated by contaminated bile, causing acute pancreatitis and chronic pancreatitis [5, 6]. Unlike biliary tract cancer, the relationship between PBM and pancreatic cancer remains unclear. Recently, Sato K et al. published a case report containing literature review of PBM and pancreatic cancer [7]. In this report, pancreatic cancer associated with congenital biliary dilatation revealed that biliary tract cancer preceded the pancreatic cancer in all metachronous multiple cancer cases. Since there are few reports of distal pancreatectomy (DP) for patients with PBM [8, 9], postoperative course after DP is largely unknown. The risk of postoperative pancreatic fistula (POPF) appears to be considerably high in patients with PBM due to constantly activated pancreatic enzymes. In addition, endoscopic sphincterotomy was performed for common bile duct stones in this patient. Pancreatic trypsinogens are also activated by refluxed duodenal juice containing enterokinase [10].

PF is a frequent complication after DP and occurs in 5–40% of resected cases [11]. Although few studies have evaluated risk factors for the development of clinically relevant POPF (CR-POPF) after DP, a recent meta-analysis reported that smoking and open DP were risk factors and that diabetes was protective factor of CR-POPF [12]. Although linear stapler is usually used in laparoscopic DP, the impact of the pancreatic stump closure technique (staple vs. direct suture) on CR-POPF was similar. Recently, pancreatic thickness to main pancreatic duct diameter ratio greater than 8 (a wide pancreas with a narrow duct) was reported to be a significant predictable factor for CR-POPF after stapled DP. In the patient in this case, no well-known risk factors were identified.

Various technical and medical ingenuities have been used to reduce the incidence rate of CR-POPF. During laparoscopic DP, division of the pancreas at the neck significantly reduced CR-POPF compared to division at the body [13]. Although simple biodegradable stapler reinforcement at the transection line did not reduce CR-POPF [14], application of fibrin glue followed by wrapping the polyglycolic acid (PGA) mesh around the remnant pancreatic stump significantly reduced the rate of CR-POPF [15]. In medical intervention, a recent systematic review with meta-analysis of randomized controlled trials elucidated the usefulness of somatostatin analog in decreasing the risk for POPF after DP [16]. However, applying the above-mentioned ingenuities could not prevent the incidence of POPF in our case.

Unfortunately, CR-POPF occurs after drain removal or drain migration away from the pancreatic stump, safety and efficacy of endoscopic transpapillary pancreatic duct stent placement have been reported [17]. In our case, an intraoperatively placed drain remained near the pancreatic stump. The drainage fluid changed to yellowish in color with bile contamination on POD9, so we decided to perform additional internal drainage and separation of bile and pancreatic juice. Although endoscopic naso-pancreatic drainage (ENPD) is more effective than ERPD in achieving complete drainage of pancreatic juice, the remnant length of the pancreatic duct was not sufficient for stable placement. Unfortunately, recurrent PA rupture occurred after drainage and separation of bile and pancreatic juice. Reconsidering our postoperative management, we should have resected the remnant pancreas at this timepoint rather than choose conservative treatment. According to the uneventful postoperative course in the previous report of DP for patients with PBM [9], we do not believe simultaneous diversion surgery and total pancreatectomy is always necessary. Anastomotic failure after pancreaticoduodenectomy (PD) causes a similar situation of a mixture of pancreatic juice, bile, and intestinal juice. CR-POPF after PD is usually well controlled under optimal drain management. In this case, we placed two drains around the pancreatic stump in the first operation. Additional drain such as Winslow drain might be effective. However, PA rupture from the GDA occurred even after the second operation with additional drains and omental filling. External drainage after DP might not be sufficient for patients with PBM and sphincterotomized papilla. Although prophylactic pancreatic stenting did not reduce CR-POPF and is not usually recommended [18], ENPD may be effective for patients with PBM and sufficient length of the remnant pancreatic duct.

POPF sometimes causes post-pancreatectomy hemorrhage, which is primarily associated with PA bleeding. In our case, we experienced PA bleeding three times. Recently, endovascular treatment has been recognized as an effective bleeding control technique with a significantly lower mortality rate than relaparotomy [19]. Stent graft placement (SGP) is superior to embolization for bleeding control and helps preserve organ perfusion. However, SGP is challenging when very fragile vessels with unsafe anchoring zones. Selective embolization with metal coils is also an effective and widely used treatment for patients with intact portal flow or collateral arterial flow [20].

Finally, the patient died of hemorrhagic shock from the portal vein (PV). Bleeding from the PV is a rare morbidity after hepatobiliary and pancreatic surgery [21]. As surgical repair of the PV is technically challenging, endovascular intervention could be considered as an alternative treatment. SGP is preferred over simple embolization to maintain portal blood flow [22]. Although we attempted percutaneous transhepatic approach, we failed to insert a guidewire due to poor intrahepatic portal blood flow.

## Conclusions

The extremely high risk of severe pancreatic fistula after distal pancreatectomy should be considered in patients with PBM and sphincterotomized papilla. In this extraordinary situation, surgeons who hesitate to resect the remnant pancreas can lose patients.

## Data Availability

Not applicable.

## Abbreviations

ALT: Alanine aminotransferase
AST: Aspartate aminotransferase
CA: Celiac artery
CHA: Common hepatic artery
CR-POPF: Clinically relevant postoperative pancreatic fistula
CT: Computed tomography
DP: Distal pancreatectomy
ERBD: Endoscopic retrograde biliary drainage
ERPD: Endoscopic retrograde pancreatic drainage
GDA: Gastroduodenal artery
IPA: Infraphrenic artery
IPMN: Intraductal papillary mucinous neoplasm
LGA: Left gastric artery
PA: Pseudoaneurysm
PBM: Pancreaticobiliary maljunction
PD: Pancreaticoduodenectomy
PGA: Polyglycolic acid
SGP: Stent graft placement
POPF: Postoperative pancreatic fistula
PV: Portal vein
SA: Splenic artery
SMA: Superior mesenteric artery
